# MIR210HG promotes cell proliferation and invasion by regulating miR-503-5p/TRAF4 axis in cervical cancer

**DOI:** 10.18632/aging.102799

**Published:** 2020-02-21

**Authors:** Ai-Hong Wang, Can-Hui Jin, Guan-Yi Cui, Hong-Yu Li, Yin Wang, Juan-Juan Yu, Rui-Fang Wang, Xiao-Yu Tian

**Affiliations:** 1 Department of Gynecologic and Obstetrics, The First Affiliated Hospital, College of Clinical Medicine, Henan University of Science and Technology, Luoyang 471000, Henan, China; 2 Department of Gynecologic and Obstetrics, The Third Affiliated Hospital of Zhengzhou University, Zhengzhou 450052, Henan, China; 3 Department of Gastrointestinal Tumor Surgery, The First Affiliated Hospital, College of Clinical Medicine, Henan University of Science and Technology, Luoyang 471000, Henan,China; 4 University Hospital, Henan University of Science and Technology, Luoyang 471000, Henan, China

**Keywords:** MIR210HG, miR-503-5p, TRAF4, cervical cancer

## Abstract

Long non-coding RNAs (lncRNAs) play important roles in the progression of cervical cancer (CC). However, the roles and underlying molecular mechanisms of lncRNAs in CC remain unclear. In the current study, we discovered a new lncRNA MIR210HG which was upregulated in CC tissues through microarray. The upregulation of MIR210HG was associated with advanced FIGO stage, metastasis, and poor prognosis in CC patients. Function assays showed that MIR210HG inhibition significantly suppressed the proliferation, invasion, and epithelial-mesenchymal transition (EMT) processes in CC and reduced tumor growth in vivo. Mechanistically, we identified that MIR210HG might serve as a competing endogenous RNA (ceRNA) of miR-503-5p to relieve the repressive effect of miR-503-5p on TRAF4 expression in CC cells. In conclusion, we demonstrated that MIR210HG promoted CC progression through regulating the MIR210HG/miR-503-5p/TRAF4 axis, indicating that MIR210HG might act as a novel insight into CC treatment.

## INTRODUCTION

Cervical cancer (CC) is one of the most common gynecological cancer in women worldwide [1, 2]. Despite substantial advances in CC treatment in the decades, the prognosis of CC patients remains unsatisfactory due to recurrence and metastasis [3, 4]. Therefore, it is imperative to discover the underlying molecular mechanisms to better understand the pathophysiology of CC.

Long non-coding RNAs (lncRNAs), a class of transcripts longer than 200 nucleotides, which characterized the progression and initiation of tumors via epigenetic, transcriptional, and post-transcriptional modulations [5, 6]. Recently, aberrantly expressed lncRNAs have been shown to play critical roles in tumor progression [7]. For example, Zhang et al showed that overexpression of MALAT1 in renal cancer was associated with advanced clinical features and poor prognosis [8]. Li et al found that lncRNA HOTTIP promoted chemoresistance of osteosarcoma cells by targeting Wnt/β-catenin [9]. He et al found that lncRNA ABHD11-AS1 promoted colorectal cancer progression through the miR-1254/WNT11 axis [10]. However, the roles and underlying mechanisms of lncRNAs in CC remain unclear.

MicroRNAs (miRNAs) are small non-coding RNAs with a size of 18–25 nucleotides, which function as post-transcriptional regulators of target mRNAs [11]. Recently, miR-503-5p was reported to be closely associated with tumor progression. For example, Xu et al showed that miR-503-5p conferred drug resistance by targeting PUMA in colorectal cancer [12]. Sun et al found that miR-503-3p induced lung cancer cells apoptosis by regulating the expression of p21 and CDK4 [12]. Park et al reported that miR-503-5p suppressed the CD97-mediated JAK2/STAT3 pathway in metastatic or paclitaxel-resistant ovarian cancer cells [13]. However, the roles and underlying mechanisms of miR-503-5p are still largely unknown.

In the present study, we analyzed the expression profile of lncRNAs in the GEO database (GSE26511) and identified MIR210HG as one of the most upregulated lncRNAs in CC tissues. Furthermore, we showed that MIR210HG served as the sponge of miR-503-5p to regulate TRAF4 expression and consequently promoted CC progression. Therefore, these findings suggested that MIR210HG could act as a novel therapeutic target for CC treatment.

## RESULTS

### MIR210HG was upregulated in CC

**Figure 1:**
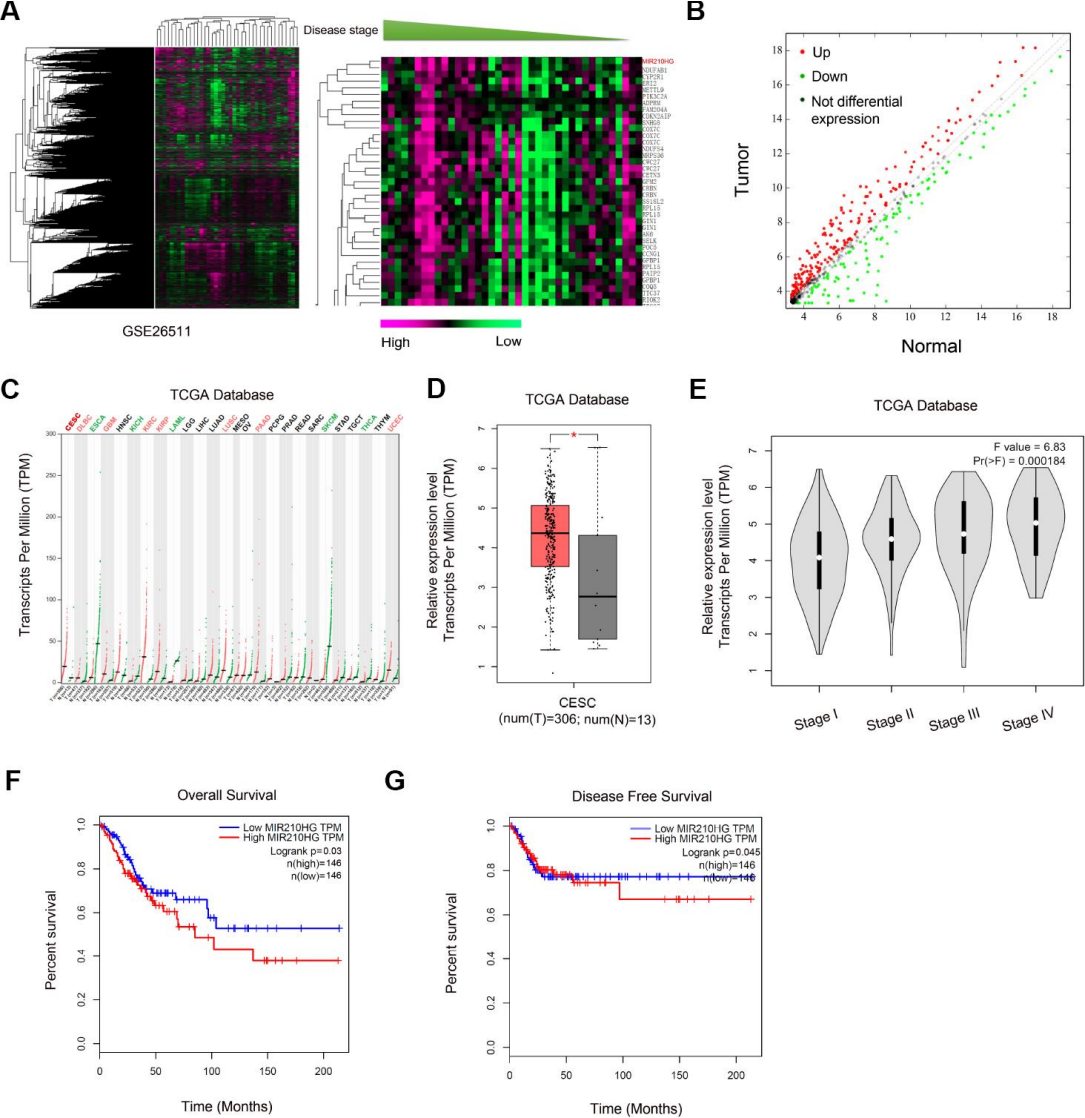
**Screening and expression of MIR210HG in CC.** (**A**, **B**) Heat map of differentially expressed lncRNAs from CC lncRNA array (GSE26511). (**B**) Volcano plot analyses of lncRNA array (GSE26511). (**C**) MIR210HG expression in tumors from TCGA database. (**D**, **E**) MIR210HG was upregulated in CESC tissues and associated with advanced pathological stage. (**F**, **G**) High MIR210HG expression was associated with poor overall survival and disease-free survival in CC patients. *P<0.05. CESC: Cervical squamous cell carcinoma and endocervical adenocarcinoma.

To identify the lncRNA participating in CC progression, we explored the GEO dataset (GSE26511). Through GEO array data analysis, we found that MIR210HG was one of the most upregulated lncRNAs in CC (Figure 1A and 1B). Subsequently, we explored MIR210HG expression in the TCGA database, results showed that MIR210HG expression was upregulated in tumor tissues, including CESC (Figure 1C and 1D). High MIR210HG expression was associated with advanced pathological stage in CC patients (Figure 1E). Furthermore, Kaplan-Meier analysis showed that high MIR210HG expression was associated with poor overall survival (OS) and disease-free survival (DFS) in CC patients (Figure 1F and 1G). Therefore, we suggested that MIR210HG might play important functions in CC development.

### MIR210HG promoted CC cells proliferation and invasion

**Figure 2:**
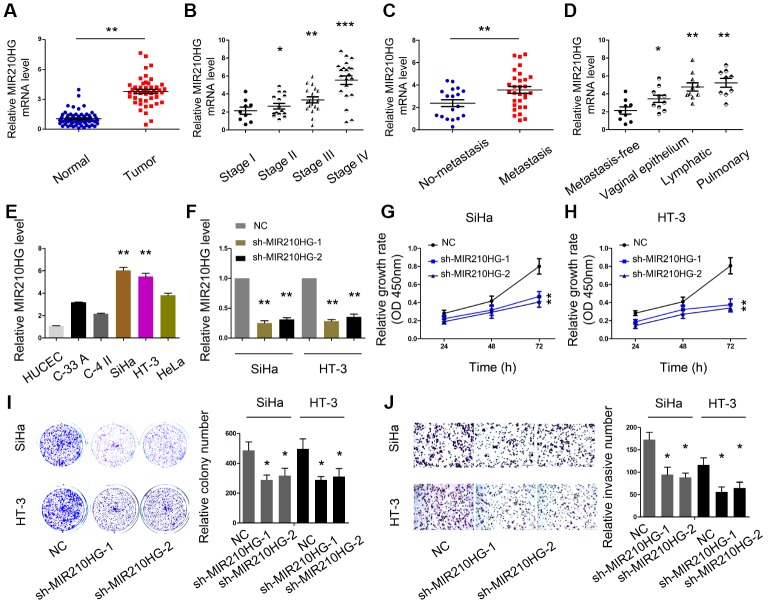
**MIR210HG promoted CC cell proliferation and invasion in vitro.** (**A**) MIR210HG was upregulated in CC tissues. (**B**–**D**) High MIR210HG expression was positively correlated with advanced FIGO stage and metastasis. (**E**) MIR210HG expression was upregulated in CC cell lines. (**F**) The knockdown efficiency of sh-MIR210HG was determined by qRT-PCR. (**G**–**I**) CCK-8 and colony formation assays were used to determine the effects of MIR210HG inhibition on CC cell proliferation abilities. (**J**) Transwell assay showed that MIR210HG inhibition reduced CC cell invasion abilities. *P<0.05.

Next, we explored the roles of MIR210HG in CC progression. We firstly measured MIR210HG expression in 67 paired CC tissues. QRT-PCR showed that MIR210HG expression was significantly upregulated and positively correlated with advanced FIGO stage and metastasis in patients (Figure 2A–2D). Furthermore, we showed that MIR210HG expression was highly expressed in CC cell lines (SiHa, C-33A, HeLa, HT-3 and C-4II) compared to HUCEC cells (Figure 2E). The SiHa and HT-3 cell lines were chosen for further experiments on account of relatively high expression of MIR210HG.

To explore the effects of MIR210HG in CC, we transfected sh-MIR210HG into SiHa and HT-3 cell lines (Figure 2F). CCK-8 and colony formation assays showed that MIR210HG suppression reduced SiHa and HT-3 cell viabilities in vitro (Figure 2G–2I). Subsequently, reduced MIR210HG expression decreased SiHa and HT-3 cell invasion abilities in vitro (Figure 2J).

### MIR210HG interacted with miR-503-5p in CC

**Figure 3:**
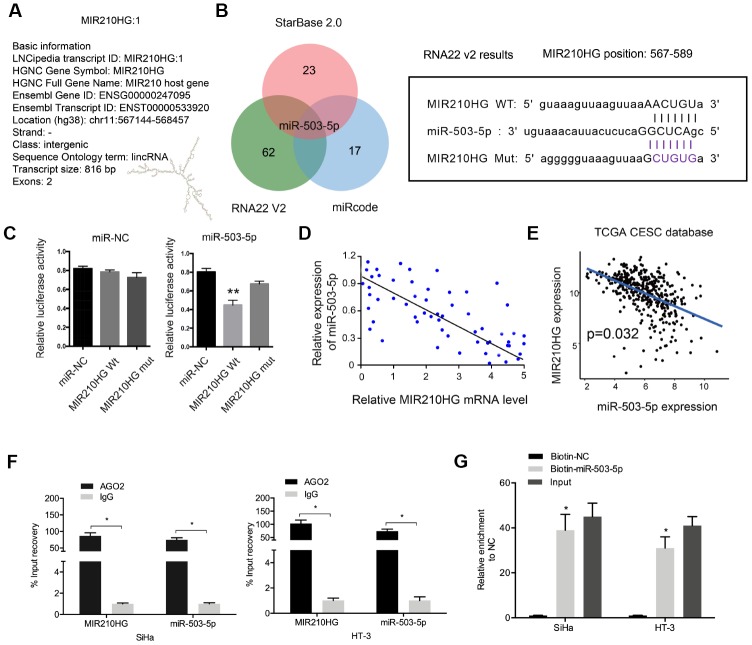
**MIR210HG interacted with miR-503-5p in CC.** (**A**) The information about MIR210HG. (**B**) Sequence alignments between miR-503-5p and seed sequence of the 3′-UTR of MIR210HG. (**C**) MiR-503-5p mimics reduced the luciferase activity of MIR210HG-Wt group. (**D**, **E**) MiR-503-5p expression was negatively correlated with MIR210HG expression in CC tissues. (**F**, **G**) RIP and pull-down assays verified the interaction between MIR210HG and miR-503-5p in CC. *P<0.05.

Recently, increasing studies reported that lncRNA might act as a miRNA “sponge” to regulate miRNA expression [14, 15]. In the present study, bioinformatics analysis (miRcode, RNA22V2, and miRcode) showed that miR-503-5p could bind to MIR210HG (Figure 3A and 3B). Dual-luciferase reporter assay showed that miR-503-5p mimics significantly reduced relative luciferase activity of MIR210HG-Wt group (Figure 3C). Correlation analysis revealed that miR-503-5p expression was negatively correlated with MIR210HG expression in CC tissues (Figure 3D). And the results were further confirmed by the TCGA database (Figure 3E). Moreover, RIP and pull-down assays further verified the interaction between MIR210HG and miR-503-5p in CC (Figure 3F and 3G). These data indicated that MIR210HG might interact with miR-503-5p in CC.

**Figure 4:**
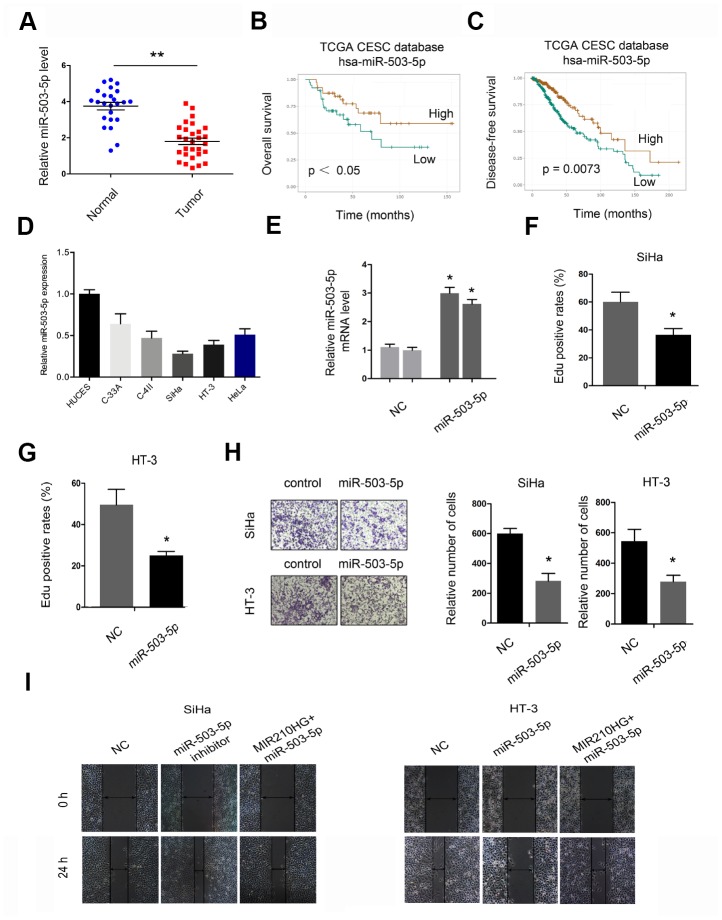
**The roles of miR-503-5p in CC progression.** (**A**, **D**) MiR-503-5p expression was downregulated in CC tissues and cell lines. (**B**, **C**) Low miR-503-5p expression was associated with poor overall survival and disease-free survival in CC patients. (**E**) The overexpression efficiency of miR-503-5p was confirmed by qRT-PCR. (**F**, **G**) MiR-503-5p mimics reduced CC cell proliferation abilities. (**H**) MiR-503-5p mimics reduced CC cell invasion abilities. (**I**) MiR-503-5p mimics abolished the effects of MIR210HG on CC cell migration abilities. *P<0.05.

Next, we explored the roles of miR-503-5p in CC. QRT-PCR showed that miR-503-5p expression was downregulated in CC tissues and cell lines (Figure 4A and 4D). Low miR-503-5p expression was associated with poor overall survival and disease-free survival in CC patients (Figure 4B and 4C). Subsequently, we transfected miR-503-5p mimics into SiHa and HT-3 cells, and the transfection efficiency was determined by qRT-PCR (Figure 4E). EdU assay showed that miR-503-5p overexpression reduced the proliferation of SiHa and HT-3 cells in vitro (Figure 4F and 4G). Transwell assay demonstrated that miR-503-5p mimics reduced SiHa and HT-3 cells invasion abilities (Figure 4H). In addition, rescue assay confirmed the MIR210HG/miR-503-5p axis in CC progression (Figure 4I).

### TRAF4 was a target gene for miR-503-5p

**Figure 5:**
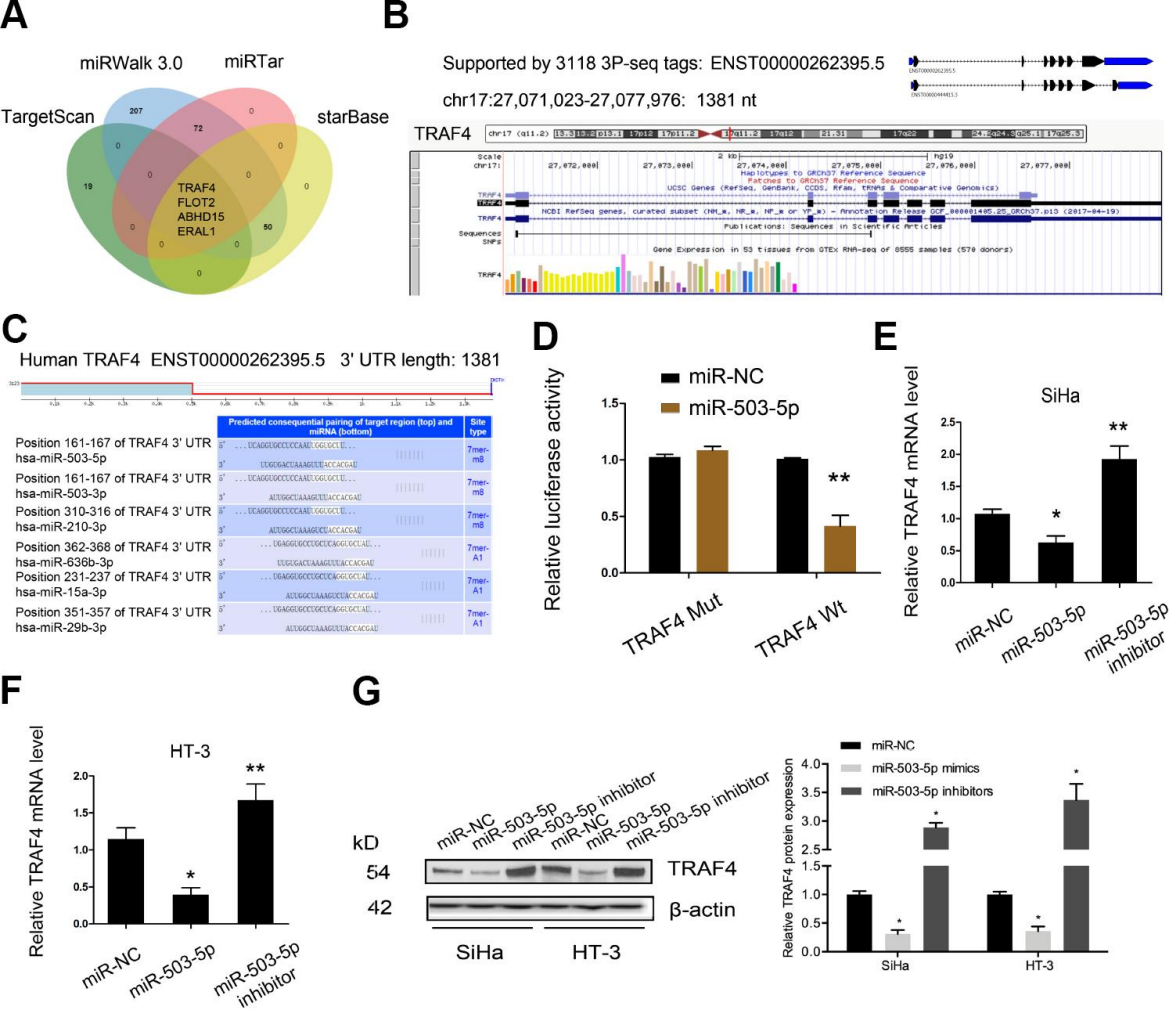
**TRAF4 was a target gene for miR-503-5p.** (**A**–**C**) MiR-503-5p target TRAF4 mRNA 3′UTR with a high score. (**D**) MiR-503-5p mimics reduced the luciferase activity of TRAF4-Wt group. (**E**–**G**) The effects of miR-503-5p on TRAF4 expression both in mRNA and protein levels. *P<0.05.

Next, we determined the downstream target genes of miR-503-5p. According to the prediction results (miRWalk 3.0, miRTar, StarBase and TargetScan), MiR-503-5p could target TRAF4 mRNA 3′UTR with a high score (Figure 5A–5C). Luciferase reporter assay confirmed the interaction between miR-503-5p and TRAF4 (Figure 5D). Subsequently, we showed that miR-503-5p mimics decreased TRAF4 expression in SiHa and HT-3 cell lines, while miR-503-5p inhibitors increased TRAF4 expression (Figure 5E–5G).

**Figure 6:**
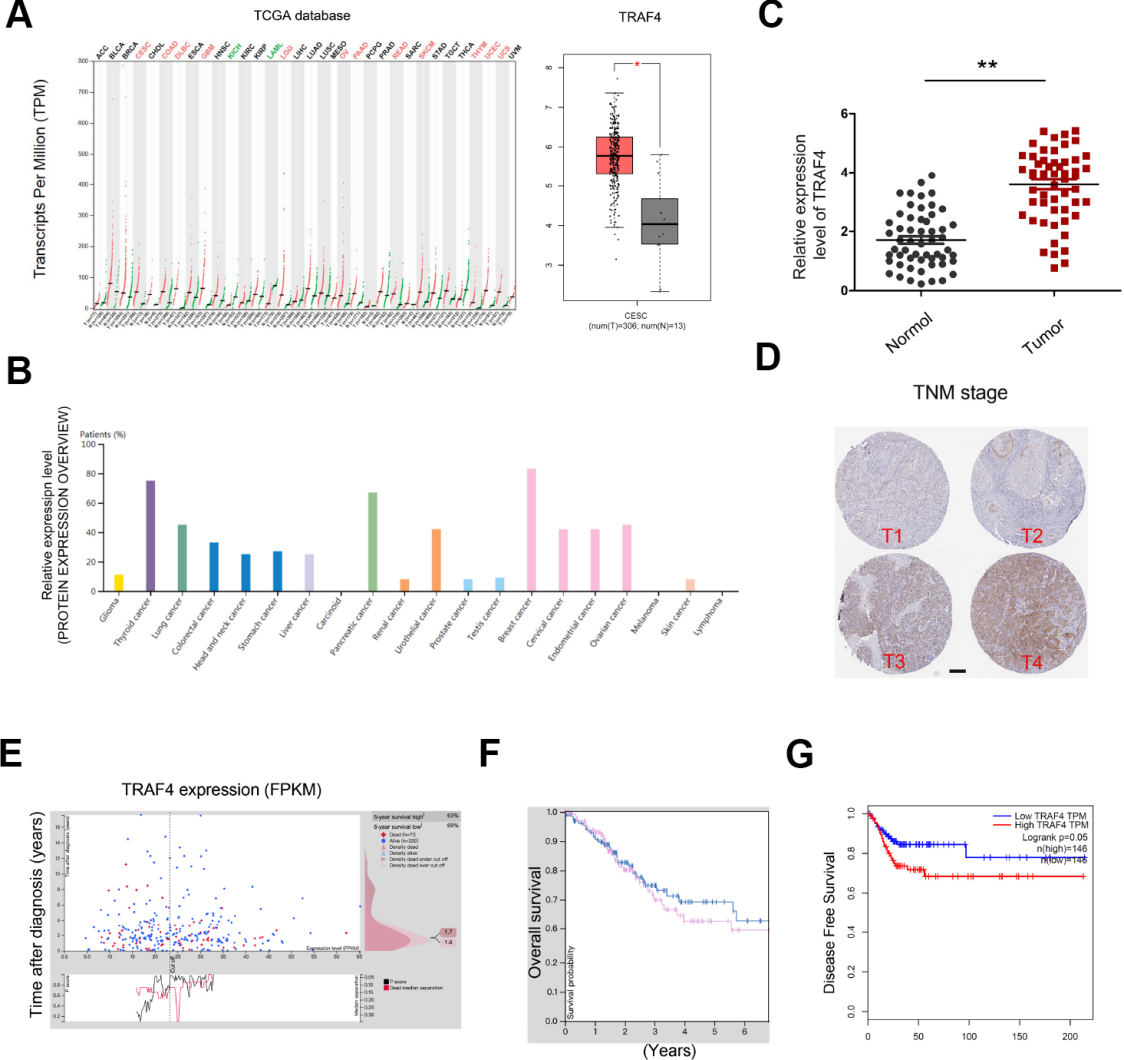
**TRAF4 expression in CC.** (**A**, **B**) TRAF4 expression in the TCGA database. (**C**) TRAF4 expression was upregulated in CC tissues. (**D**) High TRAF4 expression was associated with advanced TNM stage. (**E**–**G**) High TRAF4 expression was associated with poor overall survival and disease-free survival in CC patients. *P<0.05.

In addition, we explored the roles of TRAF4 in CC progression. TCGA database showed that TRAF4 expression was significantly upregulated in tumor tissues, especially in CESC (Figure 6A and 6B). To confirm the results, we explored TRAF4 expression in CC tissues. QRT-PCR showed TRAF4 expression was significantly upregulated and associated with advanced TNM stage of CC patients (Figure 6C and 6D). Moreover, we found that high TRAF4 expression was associated with poor prognosis in CC patients (Figure 6E–6G).

### MIR210HG/miR-503-5p/TRAF4 axis in CC

**Figure 7:**
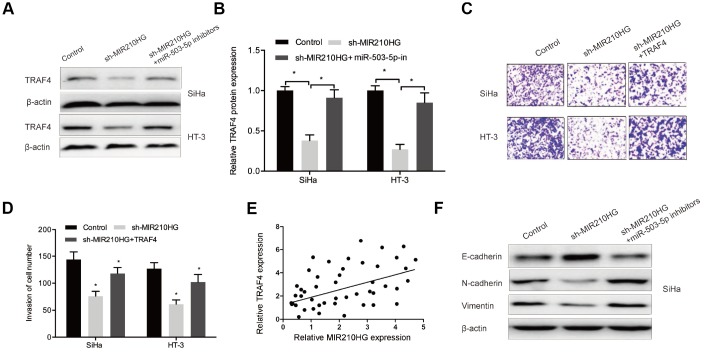
**The MIR210HG/miR-503-5p/TRAF4 axis in CC.** (**A**, **B**) MiR-503-5p inhibitors abolished the effects of MIR210HG suppression on TRAF4 expression in CC cells. (**C**, **D**) TRAF4 upregulation rescued the effects of MIR210HG suppression on CC cell invasion abilities. (**E**) MIR210HG expression was positively associated with TRAF4 expression in CC tissues. (**F**) MiR-503-5p inhibitors abolished the effects of MIR210HG suppression on EMT related gene expression in SiHa cells. *P<0.05.

To further investigate whether MIR210HG regulated CC progression through the miR-503-5p/TRAF4 axis. We firstly explored the effects of MIR210HG on TRAF4 expression in CC cells. Western blot showed that MIR210HG suppression significantly reduced TRAF4 expression in CC cells, while miR-503-5p inhibitors abolished the effects (Figure 7A and 7B). Function assay showed that the effects of MIR210HG suppression on CC cells invasion could be reversed by TRAF4 upregulation in CC cells (Figure 7C and 7D). Correlation analysis showed that TRAF4 expression was positively associated with MIR210HG expression in CC tissues (Figure 7E). Furthermore, western blot showed that MIR210HG inhibition significantly reduced the expression of Vimentin, N-cadherin and increased the expression of E-cadherin in SiHa and HT-3 cells, and the effects could be restored by miR-503-5p inhibitors (Figure 7F).

### MIR210HG promoted CC growth via miR-503-5p/TRAF4 axis in vivo

**Figure 8:**
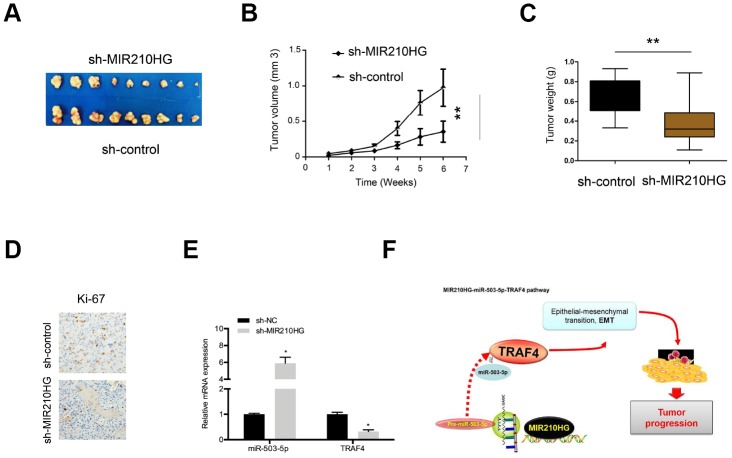
**MIR210HG suppression reduced tumor growth in vivo.** (**A**) Representative image of nude mice injected with SiHa cells. (**B**, **C**) MIR210HG suppression decreased tumor growth and weight. (**D**) MIR210HG suppression reduced Ki-67 expression in nude mice. (**E**) The effects of MIR210HG suppression on miR-503-5p and TRAF4 expression in nude mice. (**F**) The schematic diagram of the MIR210HG/miR-503-5p/TRAF4 axis in CC. *P<0.05.

In xenograft tumor model, we showed that MIR210HG suppression decreased tumor volume of nude mice as well as tumor weights compared to the control group (Figure 8A–8C). IHC showed that MIR210HG suppression reduced Ki-67 expression in nude mice (Figure 8D). Furthermore, qRT-PCR showed that MIR210HG suppression expression induced the expression of miR-503-5p and reduced the expression of TRAF4 in nude mice (Figure 8E). Taken together, we illustrated that MIR210HG promoted CC progression through regulating the miR-503-5p/TRAF4 axis (Figure 8F).

## DISCUSSION

Cervical cancer (CC) is one of the most common gynecological malignant tumors worldwide [16]. Recently, A number of studies revealed that lncRNA could be functioned as an oncogene or tumor suppressor in CC progression and development. For example, Chen et al found that upregulation of lncRNA CCAT2 was associated with metastasis and poor overall survival in CC patients [17]. Ou et al found that decreased miR-138-5p expression by lncRNA H19 promoted the proliferation of CC cells [18]. Gao et al found that lncRNA SBF2-AS1 promoted CC progression through the miR-361-5p/FOXM1 axis [19].

In the present study, through GEO array data analysis, we identified that MIR210HG was one of the most upregulated lncRNA in CC. Recently, there are some reports about the roles of MIR210HG in human cancers. For example, Wang et al found that MIR210HG could predict poor prognosis and serve as an oncogenic lncRNA in the progression of hepatocellular carcinoma [20]. Kang et al showed that MIR210HG promoted lung cancer progression through regulating methylation of CACNA2D2 promoter via binding to DNMT1 [21]. However, the roles and underlying mechanisms of MIR210HG in CC remain unclear. Herein, we revealed that MIR210HG expression was upregulated and associated with advanced clinical features and poor prognosis in CC patients. Loss-of-function assays suggested that MIR210HG inhibition reduced tumor growth, and metastasis in CC.

Increasing evidence revealed that lncRNAs could function as a miRNA sponge to regulate tumor progression [22, 23]. In the present study, we elucidated that miR-503-5p was a downstream gene of MIR210HG. Recently, various studies demonstrated that miR-503-5p might play critical roles in tumor progression [24, 25]. However, the roles and underlying mechanisms remain unknown. In this study, we showed that miR-503-5p was low expressed and associated with poor prognosis in CC patients. Function assays showed that miR-503-5p. overexpression reduced CC cell proliferation and invasion in vitro. Furthermore, the interaction between MIR210HG and miR-503-5p was confirmed in CC.

Tumor necrosis factor receptor-associated factor 4 (TRAF4) has been shown to play emerging roles in tumor metastasis, development, and chemo-resistance [26, 27]. Nevertheless, the roles of TRAF4 in CC are still unclear. In the current study, TRAF4 was discovered as a target gene of miR-503-5p in CC. Subsequently, TRAF4 was overexpressed in CC tissues and positively associated with MIR210HG expression. Moreover, rescue assays showed that the effects of MIR210HG inhibition on CC progression could be abolished by miR-503-5p inhibitors (or TRAF4 overexpression). Thus, we demonstrated that MIR210HG promoted CC progression through regulating the miR-503-5p-mediated TRAF4 axis.

Taken together, our study demonstrated an oncogenic role of MIR210HG in CC for the first time. In mechanism, we revealed that MIR210HG promoted CC progression through targeting the miR-503-5p/TRAF4 axis. Our findings demonstrated that MIR210HG might serve as a new therapeutic target in CC treatment.

## MATERIALS AND METHODS

### Patients and tissue specimens

67 paired CC tissues were collected through surgical resection from patients treated in the Third Affiliated Hospital of Zhengzhou University. No patients received any chemotherapy, immunotherapy, or radiotherapy before surgery. All these specimens were frozen in liquid nitrogen and stably stored at − 80 °C until RNA extraction. The study was conducted under the approval of the Third Affiliated Hospital of Zhengzhou University. Written informed consents were obtained from all participating patients.

### Cells culture and transfection

Normal human cervical squamous epithelial cell line (HUCEC) and human CC cell lines (SiHa, C-33A, HeLa, HT-3 and C-4II) were purchased from American Type Culture Collection (ATCC, VA, USA). Cells were cultured in Dulbecco’s modified eagle’s medium (DMEM, Gibco, USA) comprised of 10% fetal bovine serum (FBS; Thermo Fisher, Rockford, IL, USA) in a humidified incubator containing 5% CO_2_ at 37 °C.

MIR210HG was silenced by sh-MIR210HG#1, sh-MIR210HG#2 [28]. The pcDNA3.1 vector inserted with TRAF4 full sequences were used to overexpress TRAF4. The overexpression of miR-503-5p was realized by miR-503-5p mimics, and the knockdown of miR-503-5p was realized by miR-503-5p inhibitors. All plasmids were produced by GenePharma (Shanghai, China) and transfected in cells by Lipofectamine 2000 (Invitrogen, CA, USA) according to the manufacturer’s instructions.

### RNA isolation and quantitative RT-PCR

Total RNA was isolated by Trizol reagents according to the manufacturer’s instructions. cDNA was synthesized through the reverse transcription kit (Takara, Japan). Then, the mRNAs were isolated and reversed through Qiagen reverse transcription kits (Hilden, Germany). Next, we performed PCR analysis using an ABI step one real-time PCR System (Thermo Fisher, Rockford, IL, USA), the PCR reaction conditions were as follows: pre-denaturation at 95°C for 30 s and 40 cycles of denaturation at 95°C for 5 s, annealing and extension at 60°C for 30 s. Relative expression levels of lncRNA, mRNA, and miRNA were calculated by 2^-ΔΔCt^ method and normalized to the levels of GAPDH or U6. The primer sequences used were as follows: MIR210HG, forward, 5′-GCTTGGTAGAGTGTCACGCC-3′ and reverse, 5′-CATCTGACCGAGCCAGTTTG-3′ [20].

### Cell proliferation assay

Cell Counting Kit-8 (CCK-8, Beyotime, China) was used to evaluate cell proliferation. Transfected CC cells were plated in 96-well plates (2×10^3^ cells/well) and cultured for 24 h, 48 h, and 72 h followed by the addition of 10 μL CCK-8 solutions. The absorbance (450 nm) was examined with an enzyme immunoassay analyzer (Bio-Rad, CA, USA).

### Colony formation assay

Transfected CC cells were plated into 6-well plates for cultivation. 2 weeks later, PBS was used to clean the cells, which were fixed with 4% paraformaldehyde for 20 min and stained by 0.5% crystal violet solution for 15 min. The number of colonies was counted under an inverted microscope (Olympus, Tokyo, Japan).

### Transwell assay

Transfected CC cells were cultured in serum-free media and placed into the upper insert of Transwell chamber (8-μm pore size, Millipore, MA, USA) pre-coated with Matrigel (BD Biosciences, NJ, USA). The lower chamber contained DMEM medium with 10% FBS. After 24 h, the cells on the upper chambers were removed and cells on the lower compartment were fixed with ethanol and stained by 0.1% crystal violet. Then, cells were counted and imaged with an inverted microscope (Olympus, Tokyo, Japan).

### Western blot

Total protein was harvested and extracted from CC cells with RIPA buffer (Millipore, Bedford, MA, USA) and the protein concentration was calculated with a reagent kit via the Bradford method (Beyotime, China). The extracted proteins were separated by 12% SDS-PAGE and transferred to PVDF membranes (Millipore). Then, 5% skim milk powder was used to block the membranes in the room for 1 h and then the membranes were combined with primary antibodies at 4°C all night. The next day, the membranes were washed and incubated with secondary antibodies carrying HRP-conjugates. Bands were examined through an enhanced chemiluminescence kit (Thermo Fisher).

### Dual-luciferase reporter assay

MIR210HG or TRAF4 fragments containing putative miR-503-5p binding sites were respectively cloned into pmirGLO vector (Promega, Madison, WI, USA), named MIR210HG-WT or TRAF4-WT. Also, MIR210HG-MUT or TRAF4-MUT with the mutated putative miR-503-5p binding sites were constructed. Then constructed reporters were co-transfected with miR-503-5p mimics or miR-NC into cells, respectively. Relative luciferase activities were got by dual-luciferase reporter assay kit (Promega, Madison, WI, USA).

### RNA pull-down assay

The bio-labeled probe of miR-503-5p (biotin-miR-503-5p) and negative control (biotin-NC) was synthesized by Geneseed Biotech. The processes were according to the previous study [28].

### RNA immunoprecipitation (RIP) assay

RIP assay was used Magna RNA-binding protein immunoprecipitation kit (Millipore) and was according to the previous study [29]. In briefly, cells were lysed in complete RNA lysis buffer and then incubated with anti-Ago2 antibody or anti-IgG antibody (Millipore). Samples were subjected to Proteinase K buffer for the purpose of digesting proteins. The relative expression of MIR210HG and miR-503-5p was identified by qRT-PCR.

### Statistical analysis

Statistical analyses were performed by SPSS 22.0 (Chicago, IL, USA). Data were shown as means ± standard deviation (SD) from three independent repeats. The differences between groups were assessed by two-tailed Student’s t-test or one-way ANOVA with a Tukey’s post hoc test. P< 0.05 was considered statistically significant.

### Ethics approval

This research was approved by the Third Affiliated Hospital of Zhengzhou University and conducted in accordance with the principles of the Declaration of Helsinki.
